# Supplementary material for: Spatio-temporal control of mitosis using light via a Plk1 inhibitor caged for activity and cellular permeability
Source: Nat Commun. 2025 Feb 19;16:1599. doi: 10.1038/s41467-025-56746-5 (PMC11840123; doi:10.1038/s41467-025-56746-5)
Supplement: Supplementary file 1 — Supplementary Information [file 41467_2025_56746_MOESM1_ESM.pdf]

Supplementary information

Spatio-temporal control of mitosis using light via a Plk1 inhibitor caged for activity and cellular permeability

Victoria von Glasenapp[1,2], Ana C. Almeida[3], Dalu Chang[2,4], Ivana Gasic[3], Nicolas Winssinger[2,4]*, Monica Gotta[1,2]*

*To whom correspondence should be addressed:

nicolas.winssinger@unige.ch, monica.gotta@unige.ch

[1]Department of Physiology and Metabolism, Faculty of Medicine, University of Geneva, Geneva, Switzerland

[2]NCCR Chemical Biology, University of Geneva, Geneva, Switzerland

[3]Department of Molecular and Cellular Biology, Faculty of Science, University of Geneva, Geneva, Switzerland

[4]Department of Organic Chemistry, Faculty of Science, University of Geneva, Geneva, Switzerland

Supplementary Figures

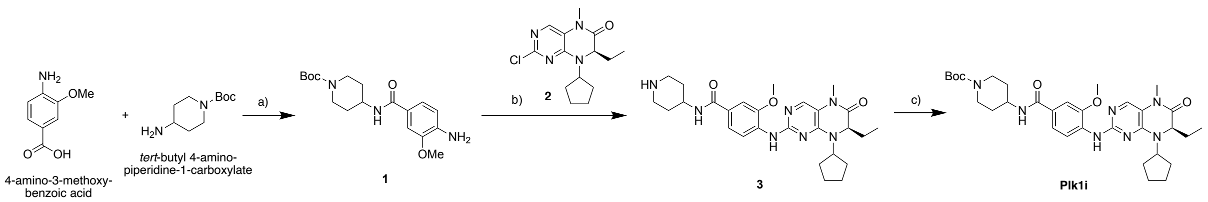

Supplementary Fig. 1: Synthesis of **Plk1i** a) HATU, Et_3N, DMF, RT, 16 h, 58% yield; b) HCl, EtOH/H_2O 1:4, 100°C, 48 h, 77% yield; c) Di-*tert*-butyl dicarbonate, DMAP, Et_3N, chloroform, RT, overnight, 90% yield.

a

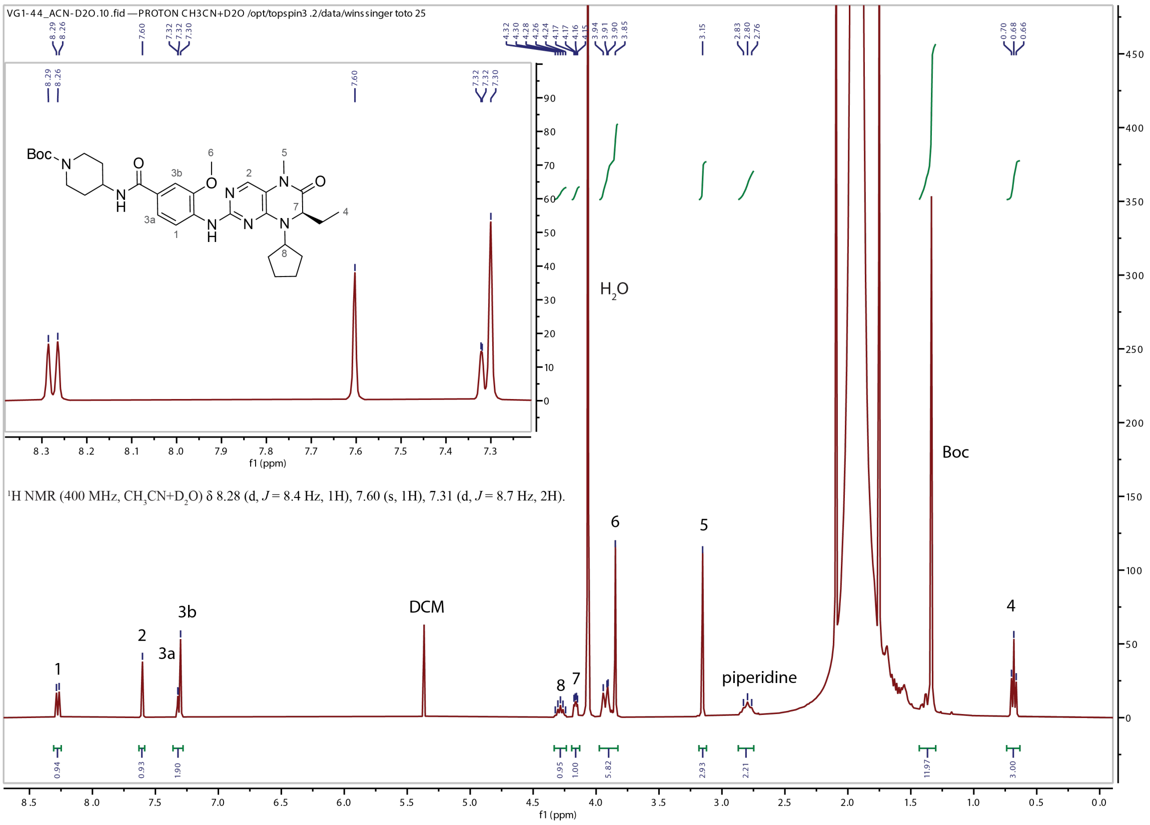

b

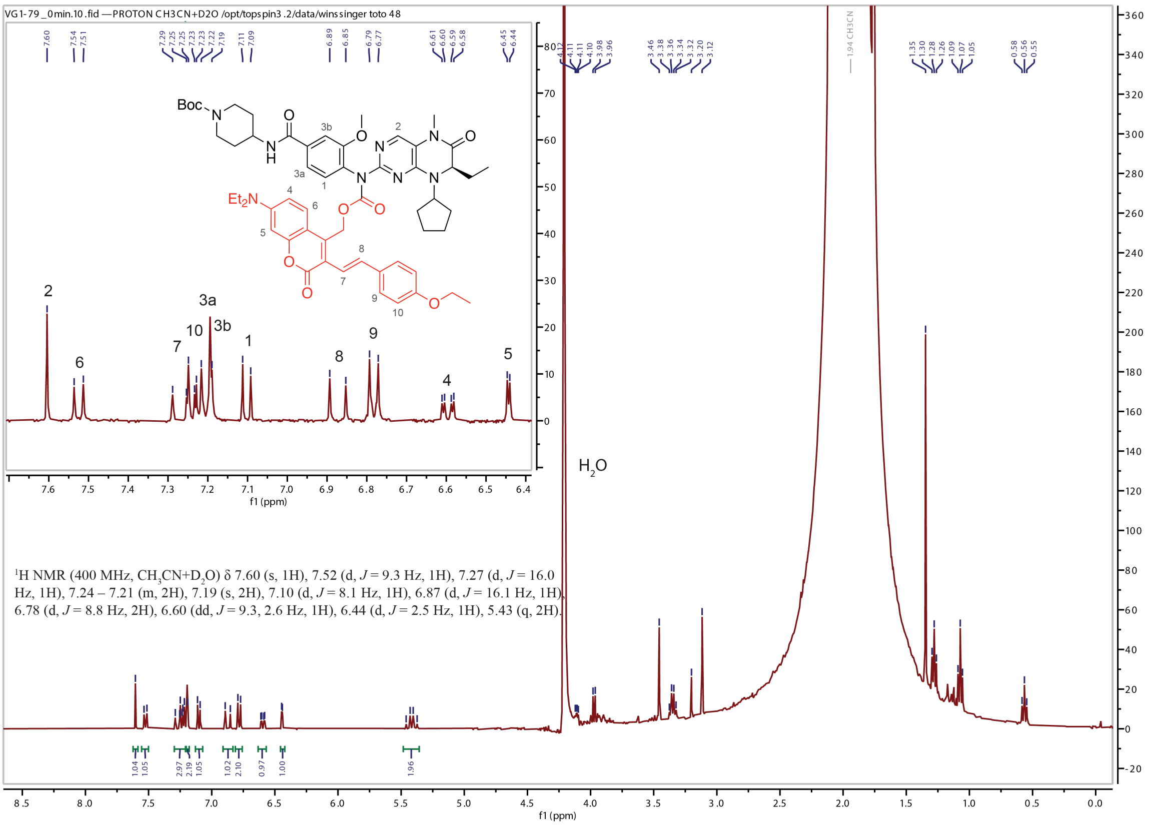

Supplementary Fig. 2: NMR spectra of **Plk1i** (a) and **cPlk1i** (b) in CH_3CN:D_2O.

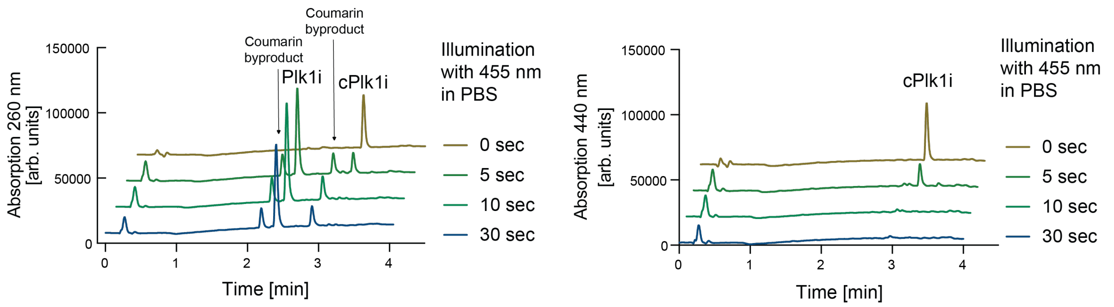

Supplementary Fig. 3: LC-MS chromatograms of cPlk1i uncaging in PBS. cPlk1i was uncaged in PBS with 455 nm light (15 mW/cm^2). The products labelled in the chromatograms (absorption 260 nm and 440 nm) are recognized thanks to their mass and their absorbance. Source data are provided as a Source Data file.

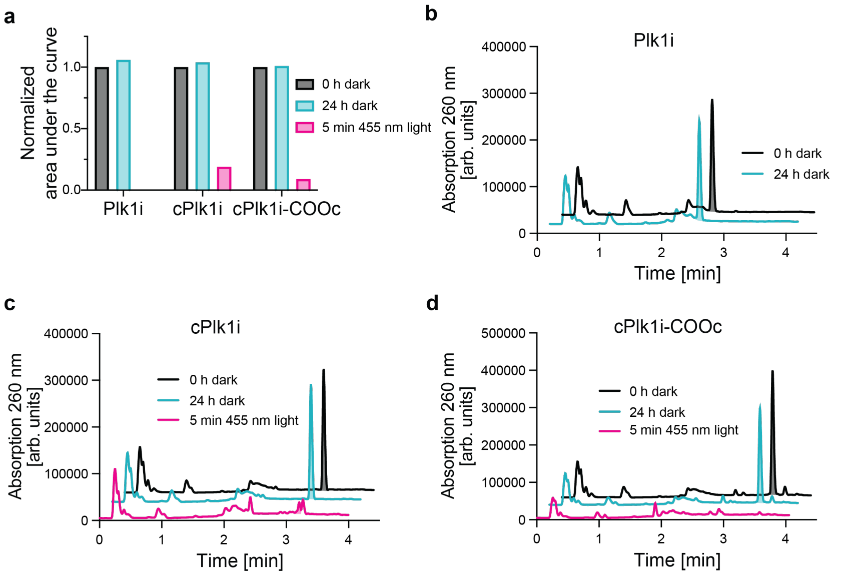

Supplementary Fig. 4: Stability in serum supplemented cell culture medium. Inhibitors (50 µM) were diluted in Fluorobrite DMEM + 10% FCS +10% DMSO for 0 to 24 hours or after illumination with 455 nm light (15 mW/cm^2). LC-MS spectra were acquired. **a)** Quantification of compound peaks. The area under the peak was normalized to the value at time 0 hours. **b-d)** LC-MS chromatograms of the indicated compounds. Source data are provided as a Source Data file.

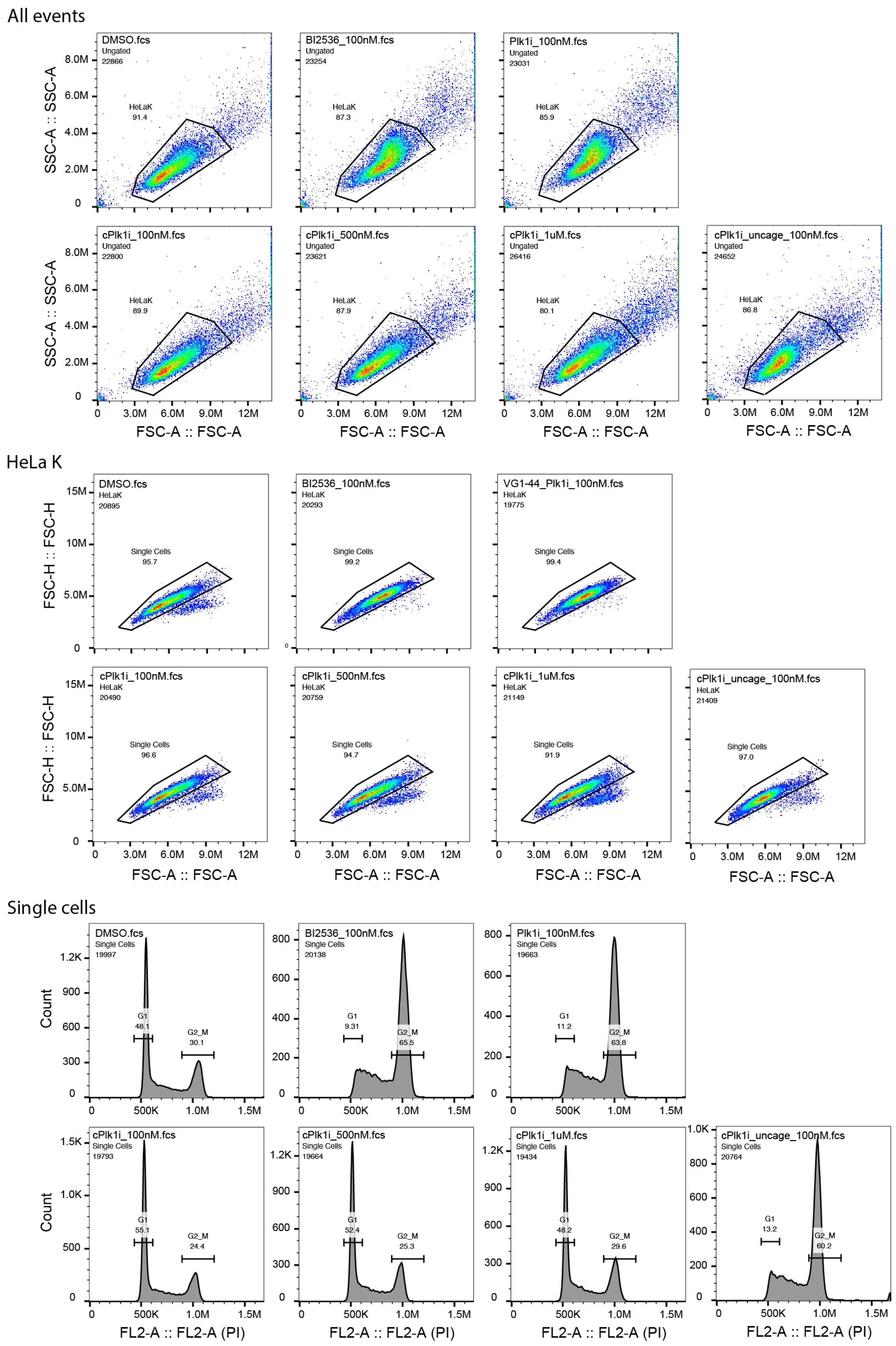

Supplementary Fig. 5: Gating strategy for cell cycle analysis.

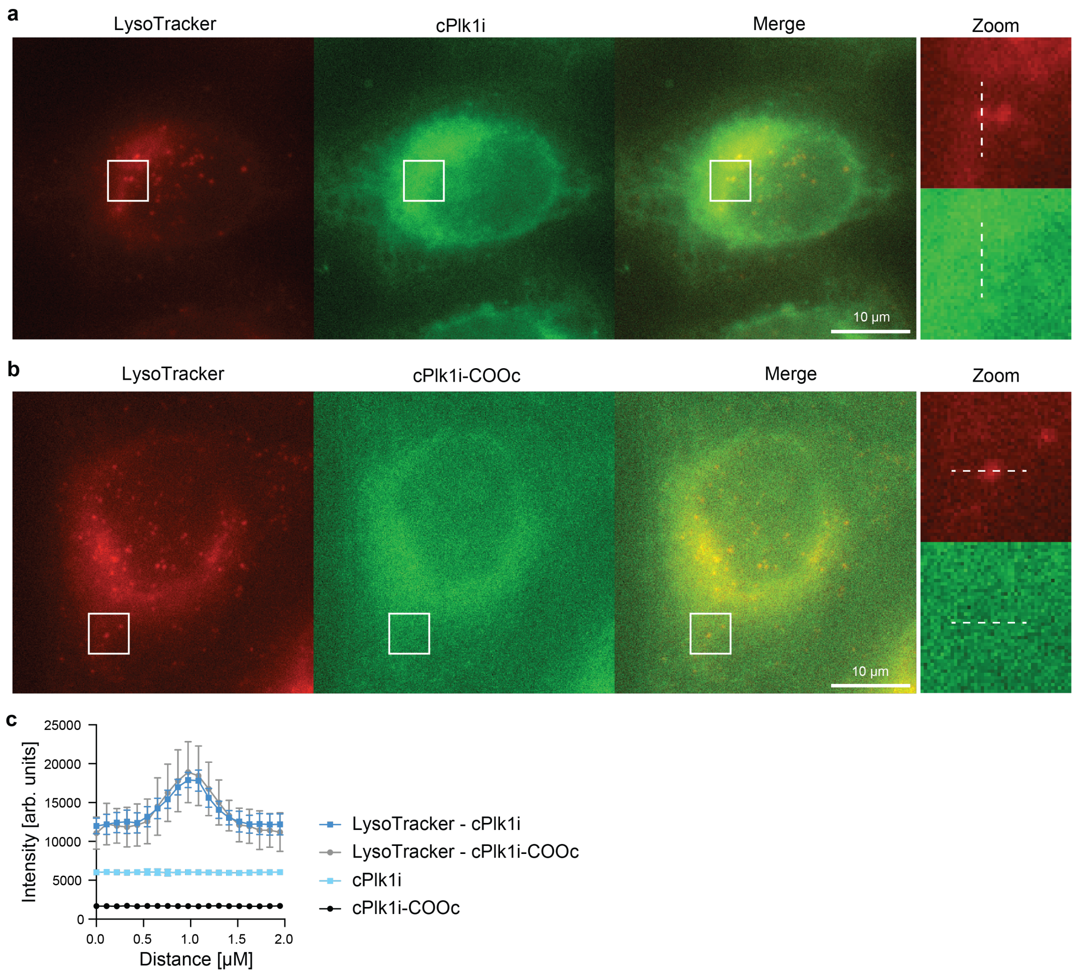

Supplementary Fig. 6: cPlk1i and cPlk1i-COOc do not colocalize with lysosomes or late endosomes. a) and **b)** HeLa K cells live stained with LysoTracker Red and treated with **cPlk1i a)** or **cPlk1i-COOc b)**. The white square shows the area of magnification on the right. The white dotted line in the magnification shows the line profile drawn to perform the quantifications shown in **c)**. The brightness in **b)** is adjusted differently compared to **a)**. **c)** Intensity of **cPlk1i** and **cPlk1i-COOc** over the line profiles of individual lysosomes. Line profiles were quantified in 9 cells for each compound. Source data are provided as a Source Data file.

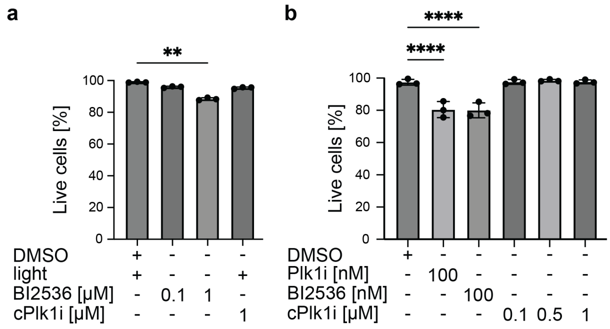

Supplementary Fig. 7: cPlk1i incubation does not result in cell death

a) Percentage of live cells measured using live-dead staining with Propidium iodide (PI) and Hoechst 33342. Cells were treated with inhibitors as indicated and illuminated with 488 nm light (5.5 mW/cm^2, 50 seconds) (+light) or not (-light) under the microscope. Live and dead cells were quantified after 18 hours of incubation. **b)** Percentage of live cells quantified as above, after 24 hours of incubation with the indicated compounds. **a, b)** Shown is the mean ± SD number of live cells. Number of cells n=275'809, N=3. Each condition was compared to the DMSO control by performing ordinary one-way ANOVA. No label means not significant (ns) p>0.6, **: p=0.0044, ****: p<0.0001 (exact p values are provided in the Source Data file). The life-dead staining data shown here and in Supplementary Fig. 16 were collected at the same time, so the data shown for the control conditions are the same. Source data are provided as a Source Data file.

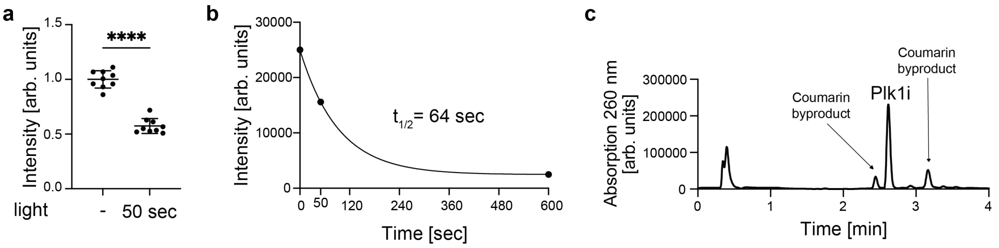

Supplementary Fig. 8: Quantification of fluorescence intensity of cPlk1i after illumination under the microscope.

a) Decrease of fluorescence intensity of **cPlk1i** (500 nM) in the entire well that has been illuminated with 488 nm light (5.5 mW/cm^2, 50 seconds) in the wide field microscope, related to Fig. 5. Datapoints correspond to technical replicates. Graph shows mean ± SD. Fluorescence intensities were compared by paired *t*-test. ****: p<0.0001. **b)** Fluorescence intensity of **cPlk1i** (1 µM) in the entire well that was illuminated with 488 nm light (5.5 mW/cm^2) in the wide field microscope. As a control, at the time corresponding to t_{600} the compound was uncaged *in vitro* with 455 nm light (15.5 mW/cm^2) for 10 minutes and then added to the cells before measuring the fluorescence intensity. **c)** LC-MS chromatogram of **cPlk1i** t_{600} before addition to the cells. Source data are provided as a Source Data file.

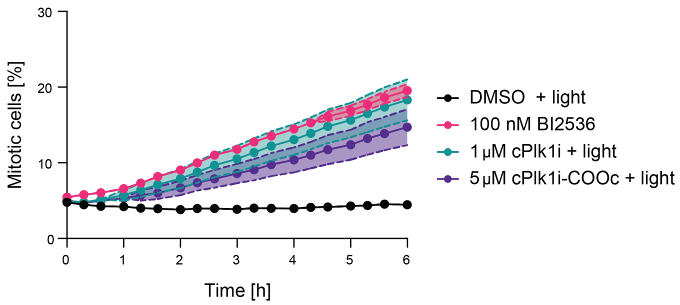

Supplementary Fig. 9: Asynchronous cells arrest in mitosis after cPlk1i and cPlk1i-COOc activation with light.
The percentage of mitotic cells compared to all cells was analyzed over 6 hours of imaging. HeLa H2B-mCherry cells were treated with the indicated inhibitors or DMSO and illuminated with 488 nm light (5.5 mW/cm^2, 50 seconds) under the microscope. Cells were imaged every 20 minutes over the course of 6 hours. Graph shows mean ± SEM, n=14'145 at 0 hours, N=3. Out of focus movies were excluded from the analysis. Source data are provided as a Source Data file.

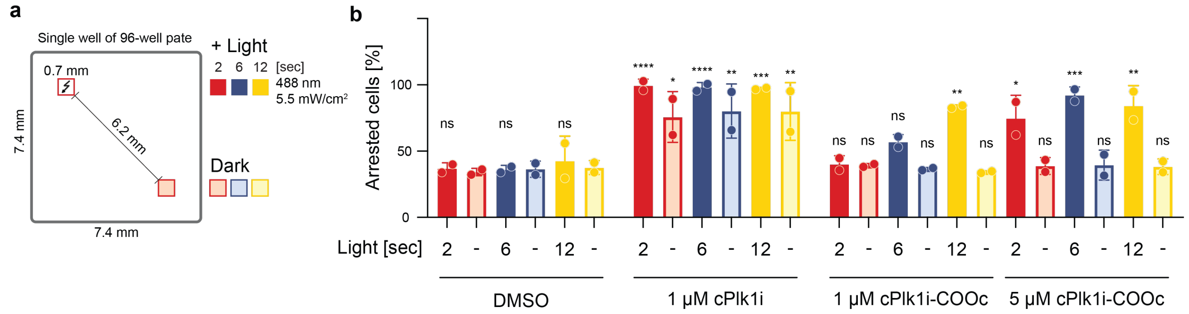

Supplementary Fig. 10: Uncaging of cPlk1i and cPlk1i-COOc
a) Schematics of exposure to light and quantification of two fields of view (FOV) in a single well. **b)** Quantification of arrested cells 120 minutes after release from Eg5 arrest. The number of arrested cells is normalized to the number of arrested cells at time 0 after monastrol release. The experimental setup was as in main Fig. 5d. DMSO 12 seconds, **cPlk1i** (1 µM) 12 seconds and **cPlk1i-COOc** (1 µM) 12 seconds is the same data as in main Fig. 5e and 6b. Shown is the mean ± SD. Number of cells n=24'262, N=2. Each condition was compared to the dark DMSO control of the respective condition by performing ordinary one-way ANOVA. ****: p<0.0001, ***: p≤0.0004, **: p≤0.0094, ns: p≥0.7333 (exact p values are provided in the Source Data file). Source data are provided as a Source Data file.

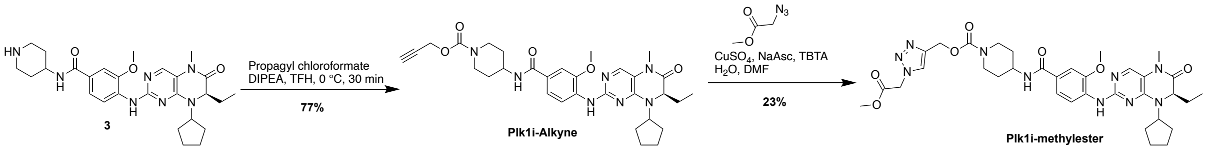

Supplementary Fig. 11: Synthesis of **Plk1i-methylester**

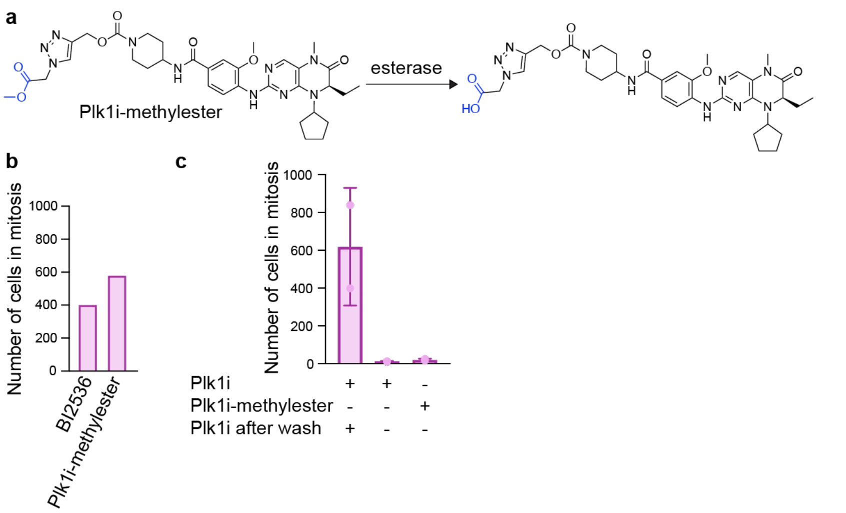

Supplementary Fig. 12: Plk1i-methylester is not retained intracellularly

a) Enzyme catalyzed ester hydrolysis of **Plk1i-methylester**. **b)** HeLa K cells treated with **Plk1i-methylester** (100 nM) for 24 hours without washing compared to BI2536 treated cells. Number of arrested cells per field of view (FOV). **c)** Cells were incubated for 3 hours with the indicated inhibitor (100 nM), washed once and incubated overnight with **Plk1i** (100 nM) as positive control or medium. Mitotic cells were counted in the FOV. The average of two FOVs is shown. Source data are provided as a Source Data file.

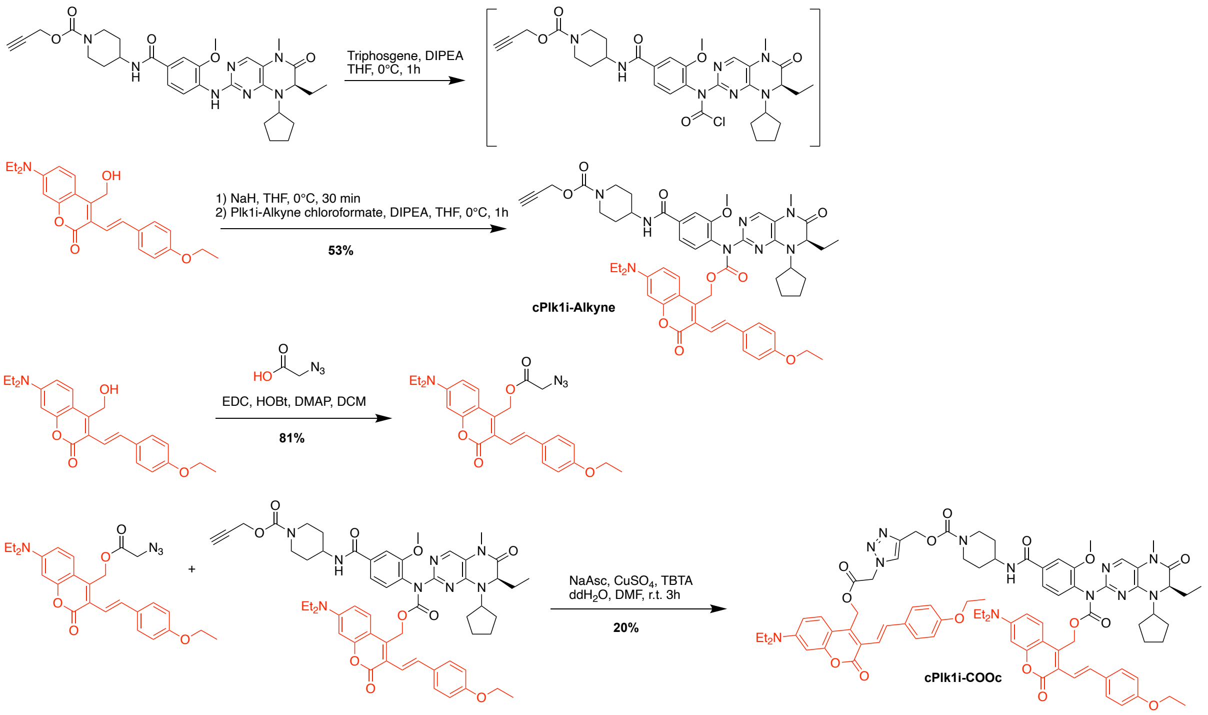

Supplementary Fig. 13: Synthesis of **cPlk1i-COOc**

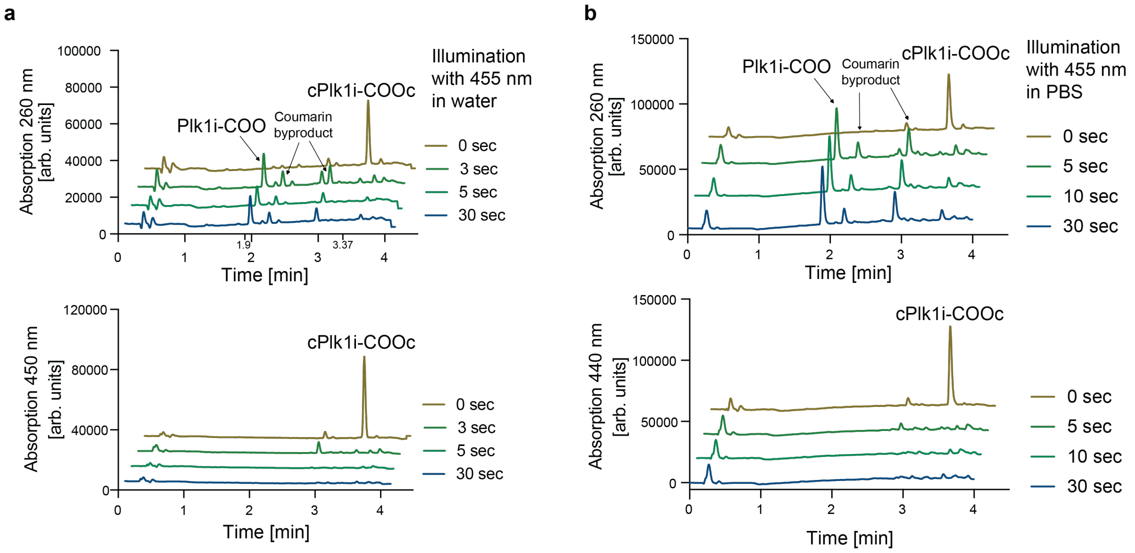

Supplementary Fig. 14: LC-MS chromatograms of cPlk1i-COOc uncaging in **a)** water and **b)** PBS with 455 nm light (15 mW/cm^2). The products labelled in the chromatograms are recognized thanks to their mass and their absorbance: **Plk1i-COO**, R_t=1.90 min, m/z 691 $[M+H]^+$, λ_{Abs}=311 nm; Coumarin byproducts, R_t=2.18 min, m/z 394 $[M+H]^+$, λ_{Abs}=380 nm, R_t=2.89 min, m/z 808 $[2M+Na]^+$, λ_{Abs}=381 nm; **cPlk1i-COO**, R_t=2.77 min, m/z 1110 $[M+H]^+$, λ_{Abs}=424 nm, **cPlk1i-COOc**, R_t=3.37 min, m/z 1486 $[M+H]^+$, λ_{Abs}=438 nm. Source data are provided as a Source Data file.

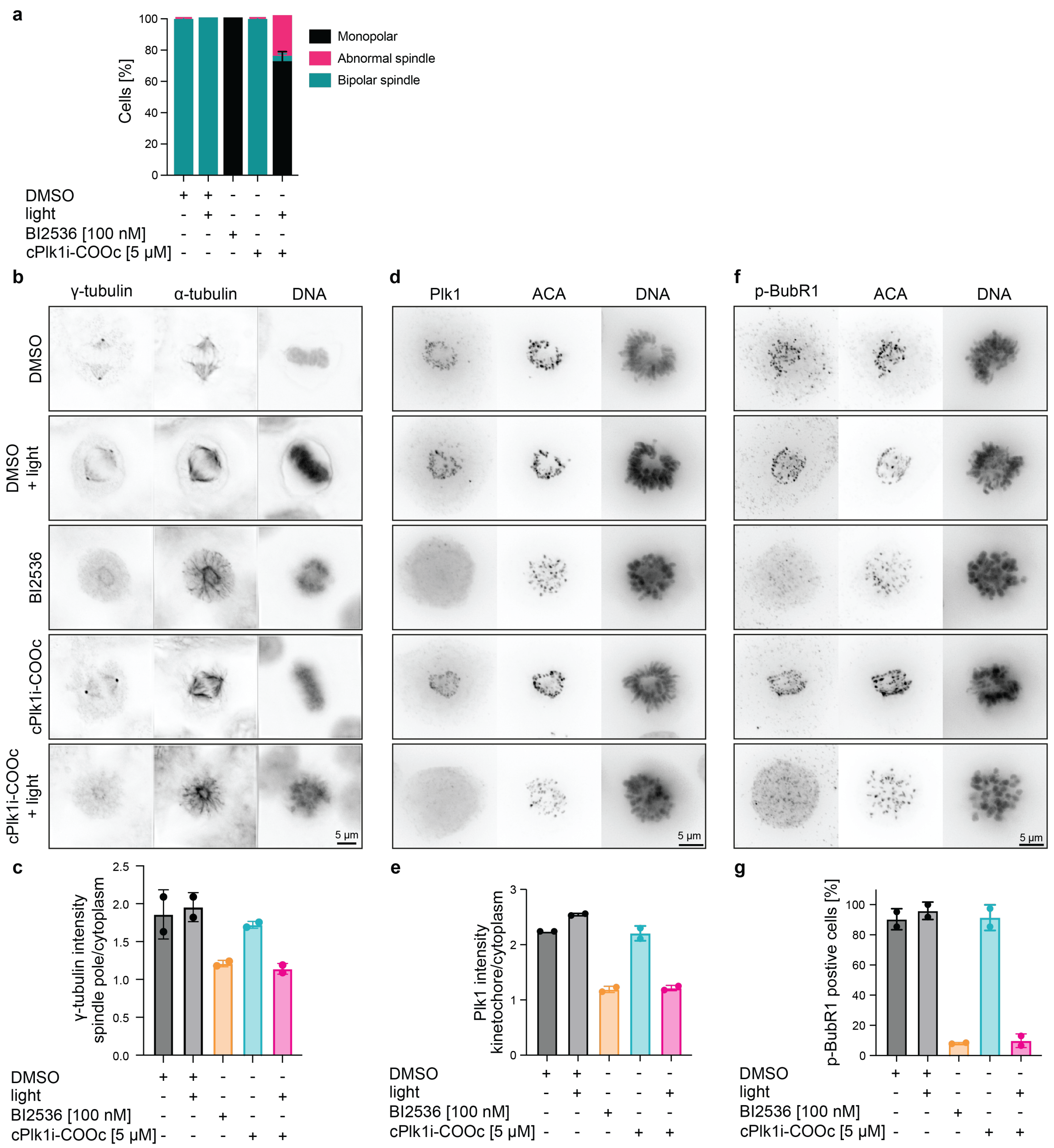

Supplementary Fig. 15: cPlki-COOc activation results in phenotypes similar to the phenotypes of cells treated with BI2536

An asynchronous population of HeLa cells was treated with DMSO or the indicated inhibitors, illuminated with 455 nm light (15.5 mW/cm^2, 3 minutes) and fixed and stained with the indicated antibodies. **a)** Quantification of mitotic spindle phenotypes n=670, N=2. **b**) Single z-planes of mitotic HeLa cells. **c)** γ-tubulin intensity at the centrosomes. Each point represents the average intensity value per experiment, mean ± SD, n=540, N=2. **d)** and **f)** Maximum intensity projection of a HeLa cell. **e)** Plk1 levels at kinetochores. Mean Plk1 intensity over cytoplasmic intensity. Each point represents the average intensity value per experiment, mean ± SD, n=163, N=2. **g)** Quantification of p676-BubR1 positive cells. Points represent percentage of positive cells per experiment. Mean ± SD, n=531, N=2. Source data are provided as a Source Data file.

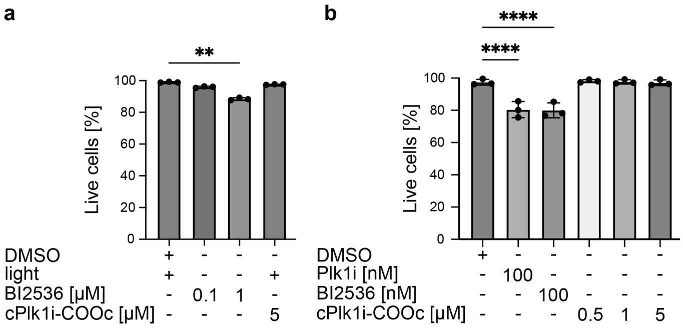

Supplementary Fig. 16: Incubation with cPlk1i-COOc does not result in cell death.
a) Percentage of live cells measured using live-dead staining with Propidium iodide (PI) and Hoechst 33342. Cells were treated with inhibitors as indicated and illuminated with 488 nm light (5.5 mW/cm^2, 50 seconds) (+light) or not (-light) under the microscope. Live and dead cells were quantified after 18 hours of incubation. **b)** Percentage of live cells quantified as above, after 24 hours of incubation with the indicated compounds. **a)**, **b)** Shown is the mean ± SD number of live cells. Number of cells n=301'885, N=3. Each condition was compared to the DMSO control by performing ordinary one-way ANOVA. No label means not significant (ns) p≥0.8, **: p=0.0044, ****: p<0.0001 (exact p values are provided in the Source Data file). The live-dead staining data shown here and in Supplementary Fig. 7 were collected at the same time, so the data shown for the control conditions are the same. Source data are provided as a Source Data file.

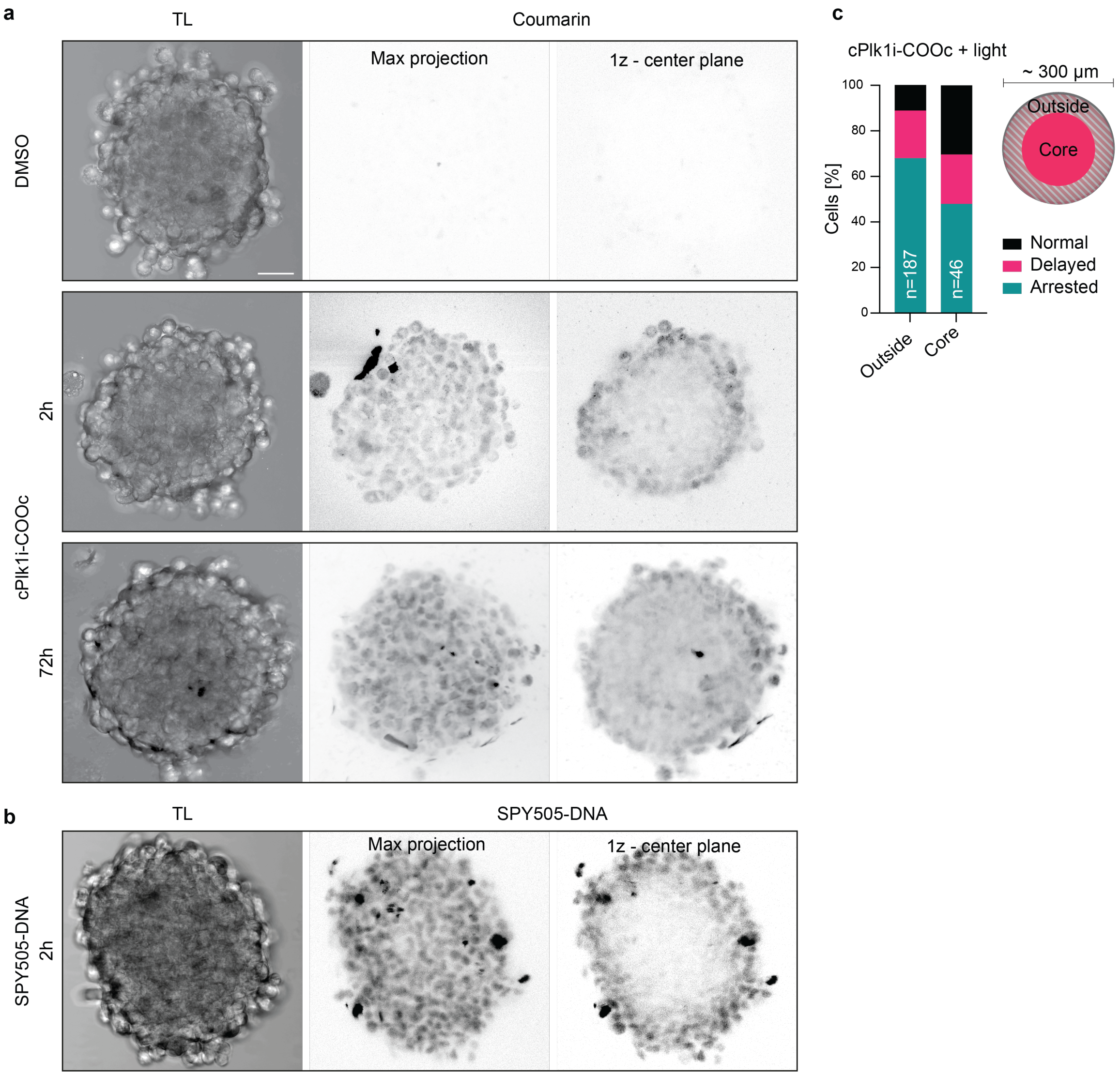

Supplementary Fig. 17: cPlk1i-COOc penetration and distribution in spheroids.
a) Representative confocal images of HeLa K spheroids treated with DMSO (0.2%) or **cPlk1i-COOc** (5 µM) at day 0 (72 hours treatment) or day 3 (2 hours treatment). Maximum intensity projection (Max projection) and the center z-plane of the spheroid are shown for coumarin fluorescence (inverted grey scale). Scale bar 50 µm. The shown fluorescence profile was observed in 6/6 spheroids (DMSO), 7/7 spheroids (2 hours **cPlk1i-COOc**) and 6/6 spheroids (72 hours **cPlk1i-COOc**). **b)** Confocal images of a 3-day HeLa K spheroid treated with SPY505-DNA for 2 hours to visualize DNA. **c)** Spatial classification of all individual cells located at the light quadrant of spheroids treated with **cPlk1i-COOc** (related to Fig. 6e). Each cell was tracked to one of the two spheroid regions - outer and inner layer. The plot illustrates the percentage of cells localized in each spheroid region against their mitotic timing. Cells were classified as "Normal" (division within 45 minutes), "Delayed" (division > 45 minutes and below 180 minutes), or "Arrested" (> 180 minutes or no exit from mitosis). Total cells analyzed: n=233. Source data are provided as a Source Data file.

Supplementary Note

In order to investigate the penetration efficiency of **cPlk1i-COOc**, we treated spheroids for 2 and 72 hours and followed the coumarin fluorescence by live microscopy. In the former case, although the middle z-plane displayed fluorescence at the center of the spheroid, the signal was not homogeneous, being higher at the outer region of the spheroid (Supplementary Fig. 17). This was significantly improved by incubating the spheroids with **cPlk1i-COOc** (5 µM) for 72 hours (added at day 0). On the other hand, treatment with the DNA dye SPY505-DNA for 2 hours led to a similar fluorescence gradient that varies along the radius of the sphere (Supplementary Fig. 17). This result suggests that the partial compound penetration observed after short incubation periods is not specific to **cPlk1i-COOc**, but rather a limitation of big 3D-cell aggregates.

Supplementary Table 1: Statistical analysis of cumulative frequency comparing medians of main Fig. 6e.

Mann-Whitney test (Wilcoxon) - two-sided - equality of medians (in red $p<0.05$)

	DMSO + Light	DMSO Dark	**cPlk1i-COOc +** Light	**cPlk1i-COOc** Dark
DMSO + Light	1.0000			
DMSO Dark	0.1324			
**cPlk1i-COOc +** Light	0.0102	0.0031	1.0000	
**cPlk1i-COOc** Dark	0.6067	0.0772	0.0074	1.0000

Supplementary Methods

Synthesis

All reagents and solvents were purchased from commercial sources and used without further purification. Anhydrous solvents were obtained by passing them through commercially available alumina column (Innovative Technology, Inc.,®VA). Analytical thin layer chromatography (TLC) was performed on silica gel 60 F_{254} (Merck). Column chromatography was carried out on silica gel (SiliaFlash® P60, SILICYCLE, 230 – 400 mesh). Reverse phase column chromatography was performed with Isolera Biotage using SNAP Cartridge KP-C18-HS of 12 g with a linear gradient of 5% ACN to 100% ACN in water with 0.1% TFA in 1 h. HPLC purification was performed with an Agilent Technologies 1260 infinity HPLC using a Thermoscientific Hypersil GOLD C-18 column (2.1 x 50 mm, 1.9 µm, 175 Å).

^{1}H and ^{13}C NMR spectra were recorded on Bruker AVANCE 3 HD NMR spectrometer 400 MHz and Bruker 500 UltraShield NMR spectrometer 500 MHz and are reported as chemical shifts (δ) in ppm relative to $CDCl_3$, $(CD_3)_2SO$ or ACN:D_20, with residual solvent peaks $CHCl_3$ (δ=7.26 ppm), $(CH_3)_2SO$ (δ=2.50 ppm), CH_3CN (δ=1.94 ppm). LC-MS spectra were recorded using a DIONEX Ultimate 3000 UHPLC coupled with a Thermo LCQ Fleet Mass Spectrometer System (electrospray ionization (ESI)) operated in positive mode. As the mobile phase 0.01% aqueous TFA solution (solution A) and 0.01% TFA in HPLC grade acetonitrile with a flow rate of 0.750 mL/min were used. Conditions for elution gradient were for method B5: 0 min to 0.5 min A:B= 95:0; 0.5 min to 3.5 min 95% A to 10% A, 3.5 min to 4 min A:B= 10:90, and for method B30: 0 min to 0.5 min A:B= 30:0; 0.5 min to 3.5 min 30% A to 0% A, 3.5 min to 4 min A:B= 0:100.

Synthesis of Plk1i

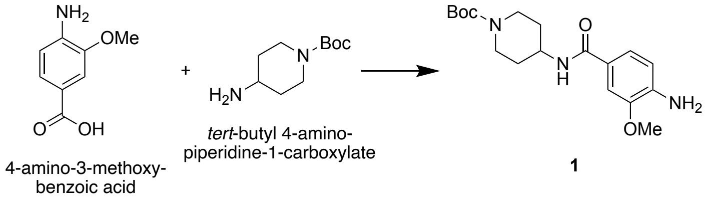

4-amino-3-methoxybenzoic acid (300 mg, 1.79 mmol, 1 eq.) was dissolved in DMF (18 mL, 0.1 M). HATU (1.09 g, 2.87 mmol, 1.6 eq.) and triethylamine (0.5 mL, 3.6 mmol, 2 eq.) were added and the reaction mixture was stirred for 15 minutes at room temperature. *Tert*-butyl 4-aminopiperidine-1-carboxylate was added. The reaction was stirred over night at room temperature. The reaction mixture was partitioned between ethyl acetate and water. The aqueous layer was extracted twice with ethyl acetate. The combined organic phases were washed 5 times with 0.1 M NaOH. The organic phase was dried over Na_2SO_4, filtered and all volatiles were removed under reduced pressure. The mixture was purified by flash column chromatography (50-100% pentane:ethylacetate) to give the product **1** as a light pink powder (363 mg, 58% yield). R_f (EtOAc)= 0.57 (UV 254 nm, stains red by ninhydrin stain upon heating).

LCMS (ESI^+, B5, R_t = 2.15 min): m/z calcd. for $C_{18}H_{27}N_3O_4$ $[M+H]^+$: 350.2, found 349.9 $[M+H]^+$, 372.1 $[M+Na]^+$, 698.6 $[2M+H]^+$.
HRMS (ESI) $[M+Na]^+$ calcd. for $C_{18}H_{26}N_3O_4Na$ 372.1899 found 372.1889.
^{1}H NMR (400 MHz, $CDCl_3$) δ 7.32 (d, *J* = 1.8 Hz, 1H), 7.10 (dd, *J* = 8.1, 1.8 Hz, 1H), 6.68 (d, *J* = 8.0 Hz, 1H), 6.05 (d, *J* = 7.8 Hz, 1H), 4.16 – 4.01 (m, 3H), 3.88 (s, 3H), 3.73 (s, 2H), 2.89 (t, *J* = 12.0 Hz, 2H), 1.98 (dd, *J* = 12.8, 3.7 Hz, 2H), 1.45 (s, 9H), 1.42 – 1.36 (m, 2H).
^{13}C NMR (101 MHz, $CDCl_3$) δ 167.0, 154.9, 147.0, 139.7, 124.3, 119.7, 113.4, 109.9, 79.8, 55.8, 47.2, 42.9, 32.4, 28.6.

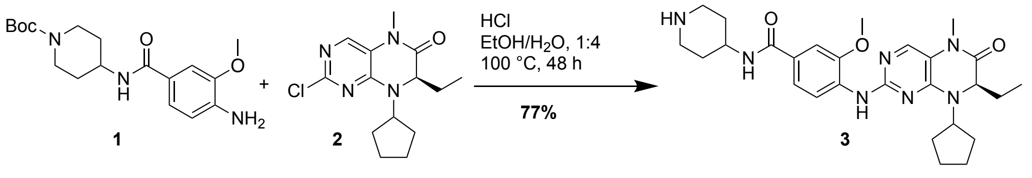

2 was synthesized according to published procedures[1]. **1** (698 mg, 2 mmol, 1 eq.) and **2** (590 mg, 2 mmol, 1 eq.) were dissolved in EtOH/H_2O 1:4 (0.2 M) and HCl (368 µL, 12 mmol, 6 eq.) was added. The mixture was left to react for 48 h under reflux. After completion of the reaction the mixture was partitioned between ethyl acetate and NaOH (1 M). The organic layer was collected and dried over Na_2SO_4, filtered, concentrated, and purified by flash column chromatography (DCM/MeOH/NH_4OH 92:7:1 to 90:9:1). The product **3** was obtained as a white solid (780 mg, 77% yield). R_f (DCM/MeOH/NH_4OH 92:7:1)= 0.87 (UV 254 nm, stained with ninhydrin).
LCMS (ESI^+, B5, R_t = 1.61 min): m/z calcd. for $C_{27}H_{37}N_7O_3$ $[M+H]^+$: 508.3, found 508.4 $[M+H]^+$, 254.9 $[M+2H]^{2+}$.
HRMS (ESI) $[M+H]^+$ calcd. for $C_{27}H_{37}N_7O_3$ 508.3036, found 508.3112.
^{1}H NMR (400 MHz, $CDCl_3$) δ 8.54 (d, *J* = 8.5 Hz, 1H), 7.68 (s, 1H), 7.59 (s, 1H), 7.42 (d, *J* = 1.9 Hz, 1H), 7.24 (d, *J* = 2.0 Hz, 1H), 6.02 (d, *J* = 8.0 Hz, 1H), 4.57 – 4.45 (m, 1H), 4.22 (dd, *J* = 7.9, 3.8 Hz, 1H), 4.11 (dtd, *J* = 11.2, 7.4, 4.0 Hz, 1H), 3.98 (s, 3H), 3.32 (s, 3H), 3.19 (dt, *J* = 12.6, 3.6 Hz, 1H), 2.81 (td, *J* = 12.2, 2.6 Hz, 1H), 2.33 – 1.93 (m, 6H), 1.92 – 1.63 (m, 8H), 1.53 (tq, *J* = 11.4, 4.0 Hz, 2H), 0.87 (t, *J* = 7.5 Hz, 3H).
^{13}C NMR (101 MHz, $CDCl_3$) δ 166.7, 163.9, 155.2, 152.4, 147.3, 138.1, 133.5, 126.3, 119.2, 116.6, 116.1, 109.2, 60.0, 58.5, 56.1, 56.1, 46.7, 45.0, 32.3, 29.9, 29.5, 28.3, 27.3, 23.8, 23.3, 9.4.

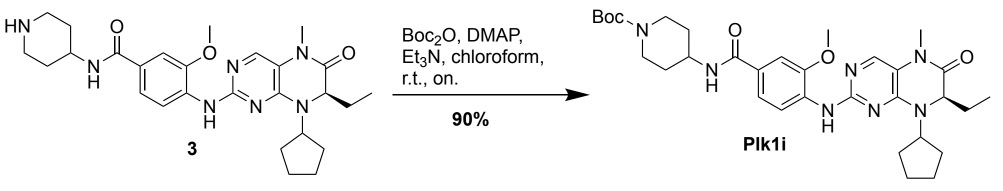

3 (157 mg, 0.31 mmol, 1 eq.) was dissolved in chloroform (0.1 M) and di-*tert*-butyldicarbonate (67.7 mg, 0.31 mmol, 1 eq.), DMAP (6.0 mg, 0.049 mmol, cat.) and triethylamine (43 µL, 0.31 mmol, 1 eq.) were added. The mixture was left to react overnight at room temperature. After completion of the reaction the mixture was partitioned between ethyl acetate and water

and dried over Na_2SO_4. After filtration the solvents were evaporated under reduced pressure and the crude was purified by flash chromatography (DCM, 5% MeOH). **Plk1i** was obtained as a white solid (167 mg, 90% yield). R_f (DCM, 5% MeOH)= 0.54 (UV 254 nm, stained with ninhydrin).
LCMS (ESI$^+$, B5, R_t = 2.39 min): m/z calcd. for $C_{32}H_{45}N_7O_5$ $[M+H]^+$: 608.4 found 608.6 $[M+H]^+$.
HRMS (ESI) $[M+H]^+$ calcd. for $C_{32}H_{45}N_7O_5$ 608.3560, found 608.3560.
^{1}H NMR (400 MHz, $CDCl_3$) δ 8.53 (d, *J* = 8.4 Hz, 1H), 7.67 (s, 1H, Ar), 7.59 (s, 1H, Ar), 7.41 (d, *J* = 1.9 Hz, 1H, Ar), 7.23 (dd, *J* = 8.5, 1.9 Hz, 1H, Ar), 6.01 (t, *J* = 6.5 Hz, 1H, CON<u>H</u>), 4.50 (p, *J* = 7.7 Hz, 1H, CH(Cp)), 4.20 (dd, *J* = 7.9, 3.7 Hz, 1H, C<u>H</u>CH_2CH_3), 4.18 – 4.02 (m, 3H, piperidine), 3.96 (s, 3H, O-CH_3), 3.31 (s, 3H, N-CH_3), 2.91 (t, *J* = 12.7 Hz, 2H, piperidine), 2.20 – 2.08 (m, 1H, Cp), 2.08 – 1.94 (m, 3H, piperidine), 1.92 – 1.60 (m, 9H, piperidine, Cp, CHC<u>H</u>$_2CH_3$), 1.50 – 1.35 (m, 11H, piperidine, Cp), 0.86 (t, *J* = 7.5 Hz, 3H, $CHCH_2C$<u>H</u>$_3$).
^{13}C NMR (101 MHz, $CDCl_3$) δ 166.7, 163.9, 155.2, 154.9, 152.4, 147.3, 138.0, 133.4, 126.4, 119.1, 116.5, 116.0, 109.2, 79.8, 77.4, 60.0, 58.5, 56.1, 47.3, 42.8, 32.4, 29.9, 29.4, 28.6, 28.3, 27.3, 23.7, 23.3, 9.3.

Synthesis of cPlk1i

Plk1i-Chloroformate

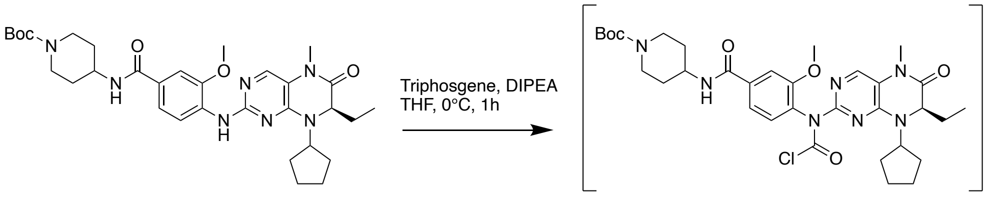

Plk1i (70 mg, 0.12 mmol, 1 eq.) was dissolved in dry THF (500 µL) and put under N_2 at 0°C. Triphosgene (103 mg, 0.35 mmol 3 eq.) was added to the reaction mixture. DIPEA (total of 102 µL, 5 eq. at 50% v/v in THF) was added dropwise to the reaction. After 1 hour the reaction went to completion as observed by LCMS in MeOH and it was quenched with saturated NH_4Cl. The aqueous phase was extracted with DCM. The organic phase was washed with brine and dried over Na_2SO_4. The solvent was evaporated under reduced pressure at room temperature. The crude was used without further purification.
LCMS (ESI$^+$, B30, R_t = 2.15 min): m/z calcd. for $C_{33}H_{44}ClN_7O_6$ $[M+H]^+$: 670.3, found 670.5 $[M+H]^+$.

Synthesis of cPlk1i

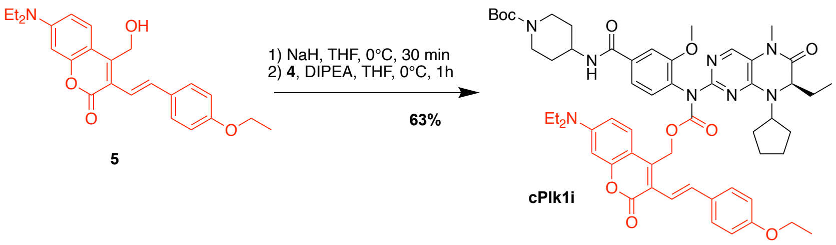

5 **cPlk1i**

The coumarin analogue **5** was synthesized as previously described[2]. The coumarin analogue (91 mg, 0.24 mmol, 2 eq.) was dissolved in dry THF (500 µL) under N_2 at 0°C and protected from light with aluminum foil. NaH (60% dispersion in mineral oil, 10 mg, 0.25 mmol, 2.2 eq) was suspended in dry THF (200 µL) added to the reaction mixture. The mixture was stirred for 30 min at 0°C. **Plk1i-chloroformate** (0.12 mmol, 1 eq.) was dissolved in THF (500 µL) and added to the coumarin reaction mixture. DIPEA (30 µL, 0.17 mmol, 1.5 eq. at 10% v/v in THF) was added dropwise to the reaction. The reaction was stirred for 1 hour at 0°C under N_2 and protected from light. The crude was partitioned between saturated NH_4Cl and DCM. The organic layer was washed with brine and dried over Na_2SO_4. The excess coumarin was removed by flash column chromatography (EtOAc 100%). The product was purified by HPLC with H_2O:ACN without TFA as a mobile phase. **cPlk1i** was obtained as a yellow solid (75 mg, 63% yield).
LCMS (ESI$^+$, B5, R_t = 3.15 min): m/z calcd. for $C_{57}H_{70}N_8O_{10}$ $[M+H]^+$: 1027.5, found 1027.25 $[M+H]^+$.
HRMS (ESI) $[M+H]^+$ calcd. for $C_{57}H_{70}N_8O_{10}$ 1027.5293, found 1049.5045 $[M+Na]^+$.
^{1}H NMR (400 MHz, DMSO) δ 8.19 (d, *J* = 7.9 Hz, 1H), 7.75 (s, 1H), 7.55 (d, *J* = 9.2 Hz, 1H), 7.44 (d, *J* = 5.1 Hz, 1H), 7.41 (s, 2H), 7.31 (d, *J* = 8.8 Hz, 2H), 7.19 (d, *J* = 8.0 Hz, 1H), 7.02 (d, *J* = 16.1 Hz, 1H), 6.88 (d, *J* = 8.8 Hz, 2H), 6.64 (dd, *J* = 9.3, 2.5 Hz, 1H), 6.54 (d, *J* = 2.5 Hz, 1H), 5.55 – 5.42 (m, 2H), 4.23 (dd, *J* = 6.7, 3.3 Hz, 1H), 4.04 (q, *J* = 7.0 Hz, 2H), 3.99 – 3.89 (m, 4H), 3.67 – 3.58 (m, 2H), 3.56 (s, 3H), 3.17 (s, 3H), 2.83 (s, 2H), 1.83 – 1.46 (m, 9H), 1.41 (s, 12H), 1.34 (t, *J* = 7.0 Hz, 6H), 1.12 (t, *J* = 7.0 Hz, 6H), 0.62 (t, *J* = 7.4 Hz, 3H).
^{13}C NMR (126 MHz, DMSO) δ 165.0, 163.8, 160.5, 158.9, 154.8, 154.7, 154.3, 153.7, 153.5, 151.2, 150.4, 142.7, 137.0, 135.0, 133.8, 132.4, 130.4, 129.1, 128.8, 128.3, 127.3, 120.3, 120.0, 119.4, 119.2, 117.6, 115.1, 115.0, 111.3, 109.6, 108.1, 96.9, 79.1, 63.5, 62.6, 61.3, 61.1, 60.2, 56.5, 56.0, 47.1, 44.5, 31.8, 29.5, 28.6, 28.2, 27.8, 27.2, 26.8, 23.8, 23.5, 15.1, 12.9, 8.7.

Synthesis of cPlk1i-COOc

Synthesis of Plk1i-Alkyne

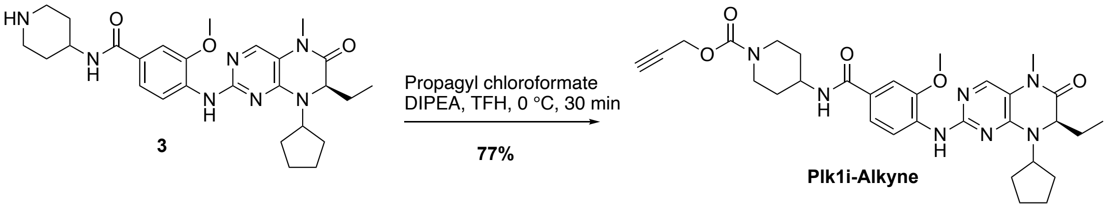

3 **Plk1i-Alkyne**

3 (200 mg, 0.39 mmol, 1 eq.) was dissolved in THF (4 mL, 0.1 M) and put under N_2 at 0°C. Propargyl chloroformate (38 µL, 0.39 mmol, 1 eq.) was added to the reaction followed by DIPEA (102 µL, 0.59 mmol, 1.5 eq.). The reaction was stirred for 30 min until completion of the reaction. The reaction was quenched with saturated NH_4Cl and the aqueous phase was extracted with DCM. The organic phase was washed with brine and dried over Na_2SO_4. The crude was purified by flash column chromatography (100% DCM to 95% DCM, 5% MeOH). R_f (DCM, 5% MeOH) = 0.46 (UV 254 nm, ninhydrin). **Plk1i-Alkyne** was obtained as a white solid (179 mg, 77% yield).
LCMS (ESI^+, B5, R_t = 2.12 min): m/z calcd. for $C_{31}H_{39}N_7O_5$ $[M+H]^+$: 590.3, found 590.39 $[M+H]^+$.
HRMS (ESI) $[M+H]^+$ calcd. for $C_{31}H_{39}N_7O_5$ 590.3091, found 590.3090.
^1H NMR (400 MHz, $CDCl_3$) δ 8.50 (d, *J* = 8.4 Hz, 1H), 7.72 (s, 1H), 7.66 (s, 1H), 7.41 (d, *J* = 1.9 Hz, 1H), 7.23 (dd, *J* = 8.5, 1.9 Hz, 1H), 6.01 (d, *J* = 7.8 Hz, 1H), 4.70 (d, *J* = 2.4 Hz, 2H), 4.57 – 4.42 (m, 1H), 4.25 – 4.20 (m, 1H), 4.20 – 4.07 (m, 2H), 3.97 (s, 3H), 3.32 (s, 3H), 3.00 (s, 2H), 2.47 (t, *J* = 2.4 Hz, 1H), 2.21 – 1.93 (m, 4H), 1.92 – 1.58 (m, 9H), 1.54 – 1.39 (m, 2H), 0.87 (t, *J* = 7.5 Hz, 3H).
^{13}C NMR (101 MHz, $CDCl_3$) δ 166.7, 163.8, 154.8, 154.5, 152.5, 147.6, 136.9, 133.2, 126.6, 119.0, 116.5, 116.4, 109.3, 78.6, 77.4, 74.7, 60.1, 58.7, 56.1, 53.6, 53.2, 47.1, 43.3, 32.3, 29.8, 29.4, 28.3, 27.4, 23.7, 23.3, 9.3.

Cu(I)-catalyzed azide-alkyne cycloaddition: Click reaction general method

To a solution of sodium ascorbate (20 µL, 500 mM in H_2O, 1.3 eq.) and $CuSO_4$ (10 µL, 400 mM in H_2O, 0.5 eq.) TBTA (1 mg, 5.3 eq.) was added in a microcentrifuge tube and diluted with 22 µL of water and 100 µL DMF. Then, the alkyne inhibitor (50 µL, 150 mM in DMF, 1 eq.) was added followed by the respective azide (50 µL, 150 mM in DMF, 1 eq.). The mixture was shaken at room temperature for 3 h to 18 h. The crude was diluted with DMF and purified by HPLC.

Methyl-2-azidoacetate[3]

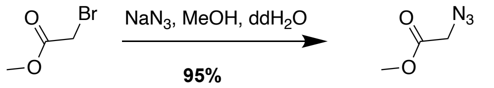

Methyl 2-bromo acetate (2.5 mL, 27 mmol, 1 eq.) was dissolved in methanol (2 mL). Sodium azide (2.2 g, 33.8 mmol, 1.25 eq.) was suspended in H_2O (2 mL) and the solution was added to the reaction at room temperature. The reaction was stirred for 20 min at room temperature and then heated to 80°C for 2 h. After cooling down to room temperature methanol was evaporated under reduced pressure. The residue was poured into H_2O and extracted twice with diethyl ether. The organic phase was dried over Na_2SO_4, filtered, and concentrated under reduced pressure. The product was obtained as a clear oil (1.8 mL, 95% yield). ^1H NMR (400 MHz, $CDCl_3$) δ 3.87 (s, 2H), 3.79 (s, 3 H), ^{13}C NMR (101 MHz, $CDCl_3$) δ 168.9, 52.7, 50.3. In agreement with the literature[3].

Plk1i-methylester

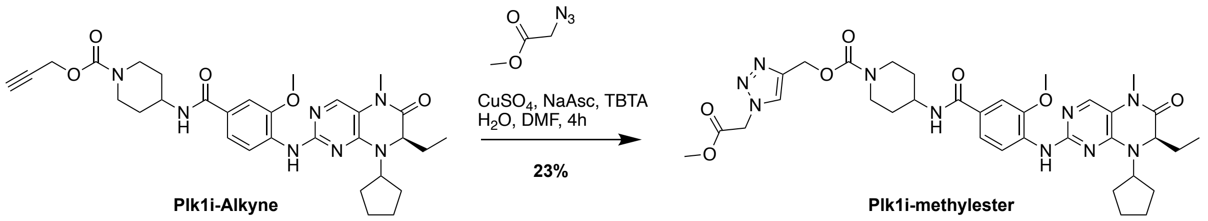

The compound was synthesized with **Plk1i-Alkyne** (3.6 mg, 6.1 µmol) and methyl-2-azidoacetic acid as starting materials following the general procedure of the click reaction. The reaction was completed after 4 hours. The product was purified by HPLC. **Plk1i-methylester** was obtained as a white solid after lyophilization (1.0 mg, 23% yield).
LCMS (ESI$^+$, B5, R_t = 2.02 min): m/z calcd. for $C_{34}H_{44}N_{10}O_7$ $[M+H]^+$: 705.3, found 705.3 $[M+H]^+$.
HRMS (ESI) $[M+H]^+$ calcd. for $C_{34}H_{44}N_{10}O_7$ 705.3573, found 705.3546.
^{1}H NMR (400 MHz, $CDCl_3$) δ 10.20 (s, 1H), 7.80 (s, 1H), 7.74 (d, *J* = 8.3 Hz, 1H), 7.44 (s, 1H), 7.38 (s, 1H), 7.22 (dd, *J* = 8.2, 1.9 Hz, 1H), 6.09 (d, *J* = 7.8 Hz, 1H), 5.26 (d, *J* = 9.1 Hz, 2H), 5.18 (d, *J* = 3.0 Hz, 2H), 4.35 (dd, *J* = 6.9, 3.2 Hz, 1H), 4.29 – 4.11 (m, 4H), 3.92 (s, 3H), 3.80 (s, 3H), 3.29 (s, 3H), 3.02 – 2.89 (m, 2H), 2.12 – 1.98 (m, 4H), 1.89 – 1.65 (m, 4H), 1.65 – 1.50 (m, 4H), 1.48 – 1.34 (m, 2H), 0.86 (t, *J* = 7.4 Hz, 4H).
^{13}C NMR (101 MHz, $CDCl_3$) δ 166.7, 166.3, 162.5, 155.1, 153.1, 152.0, 149.9, 143.9, 132.1, 129.0, 125.4, 123.4, 123.0, 118.2, 115.8, 110.2, 61.3, 61.2, 58.3, 56.1, 53.1, 50.7, 47.3, 43.2, 32.0, 31.6, 28.9, 28.6, 28.4, 27.9, 23.3, 23.1, 8.4.

Chloroformate formation of Plk1i-Alkyne

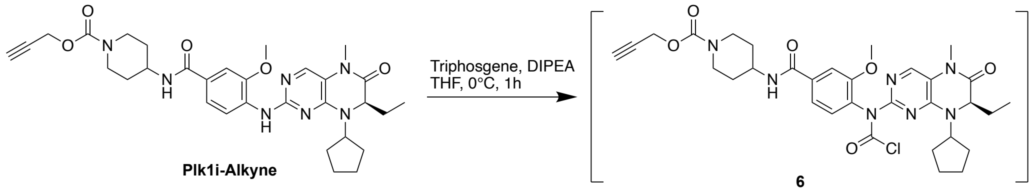

Plk1i-Alkyne (70 mg, 0.12 mmol, 1 eq.) was dissolved in dry THF (500 µL) and put under N_2 at 0°C. Triphosgene (105 mg, 0.35 mmol 3 eq.) was added to the reaction mixture. DIPEA (total of 102 µL 5 eq. at 50% v/v in THF) was added dropwise to the reaction. After 1 hour the reaction went to completion as observed by LCMS in MeOH and it was quenched with saturated NH_4Cl. The aqueous phase was extracted with DCM. The organic phase was washed with brine and dried over Na_2SO_4. The solvent was evaporated under reduced pressure at room temperature. The crude was used without further purification.

Caging Plk1i-Alkyne

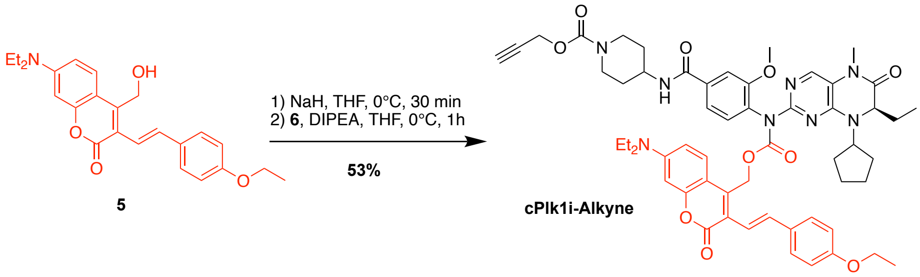

5

The coumarin analogue **5** (93 mg, 0.24 mmol, 2 eq.) was dissolved in dry THF (500 µL) under N_2 at 0°C and protected from light with aluminum foil. NaH (60% dispersion in mineral oil, 10 mg, 0.26 mmol, 2.2 eq) was suspended in dry THF (200 µL) added to the reaction mixture. The mixture was stirred for 30 min at 0°C. The chloroformate activated inhibitor **Plk1i-Alkyne** (0.12 mmol, 1 eq.) was dissolved in THF (500 µL) and added to the coumarin reaction mixture. DIPEA (30 µL, 0.17 mmol, 1.5 eq. at 10% v/v in THF) was added dropwise to the reaction. The reaction was stirred for 1 hour at 0°C under N_2 and protected from light. The solvent was then evaporated under reduced pressure. The crude mixture was purified by flash column chromatography (EtOAc 100%) and further by HPLC with H_2O:ACN without TFA as a mobile phase. **cPlk1i-Alkyne** was obtained as a yellow solid (63 mg, 53% yield).
LCMS (ESI$^+$, B5, R_t = 3.00 min): m/z calcd. for $C_{56}H_{64}N_8O_{10}$ $[M+H]^+$: 1009.5, found 1009.1 $[M+H]^+$.
HRMS (ESI) $[M+H]^+$ calcd. for $C_{56}H_{64}N_8O_{10}$ 1009.4824, found 1009.4798.
^{1}H NMR (500 MHz, $CDCl_3$) δ 7.87 (s, 1H), 7.77 (d, *J* = 9.2 Hz, 1H), 7.53 (d, *J* = 16.0 Hz, 1H), 7.25 – 7.21 (m, 3H), 7.17 (d, *J* = 8.0 Hz, 1H), 7.11 (dd, *J* = 8.0, 1.7 Hz, 1H), 6.88 (d, *J* = 16.1 Hz, 1H), 6.81 (d, *J* = 8.7 Hz, 2H), 6.62 (d, *J* = 9.2 Hz, 1H), 6.50 (s, 1H), 5.78 (s, 1H), 5.55 (s, 2H), 4.71 (d, *J* = 2.5 Hz, 2H), 4.17 (dd, *J* = 7.0, 3.4 Hz, 1H), 4.03 (q, *J* = 7.0 Hz, 2H), 3.54 (s, 3H), 3.42 (q, *J* = 7.1 Hz, 4H), 3.27 (s, 3H), 2.95 (s, 2H), 2.48 (t, *J* = 2.4 Hz, 1H), 1.96 (dd, *J* = 12.7, 3.6 Hz, 2H), 1.88 (ddd, *J* = 14.4, 7.4, 3.3 Hz, 2H), 1.63 (dd, *J* = 14.5, 7.3 Hz, 2H), 1.52 (dd, *J* = 18.6, 9.0 Hz, 3H), 1.42 (t, *J* = 7.0 Hz, 3H), 1.38 – 1.32 (m, 4H), 1.30 – 1.23 (m, 2H), 1.21 (t, *J* = 7.1 Hz, 6H), 0.75 (t, *J* = 7.5 Hz, 3H).
^{13}C NMR (126 MHz, $CDCl_3$) δ 166.3, 163.7, 161.3, 159.1, 155.4, 154.8, 154.4, 153.2, 151.8, 150.2, 141.4, 135.9, 134.3, 131.7, 130.5, 129.1, 128.3, 126.9, 119.0, 118.6, 118.6, 114.8, 114.7, 111.0, 109.7, 108.7, 97.4, 78.6, 74.7, 63.6, 62.6, 61.6, 60.2, 55.7, 53.2, 47.3, 45.1, 43.2, 31.9, 31.8, 28.4, 28.3, 28.2, 27.3, 23.1, 22.9, 15.0, 12.6, 8.7.

Synthesis of Coumarin-COO-N_3

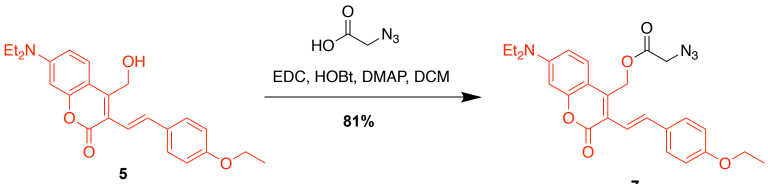

5
7

The coumarin analogue **5** (58 mg, 0.15 mmol, 1 eq.) was dissolved in DCM (1.5 mL) and azido acetic acid (22 mg, 0.22 mmol, 1.5 eq.) was added. EDC (42 mg, 0.22 mmol, 1.5 eq.), DMAP

(4 mg, 0.03 mmol, 0.2 eq.) and HOBt (34 mg, 0.22, 1.5 eq.) were added to the reaction mixture under stirring. The reaction was stirred for 1 hour under N_2 at room temperature. Then the reaction was concentrated and extracted with ethylacetate and 0.1 M HCl. The organic layer was collected and washed with brine. The organic layer was dried over Na_2SO_4. The crude mixture was purified by flash column chromatography (Pentane:EtOAc 1:1). R_f (Pentane:EtOAc 1:1) = 0.72 (UV 365 nm). The product **7** was obtained as a yellow solid (58 mg, 81% yield).

LCMS (ESI$^+$, B5, R_t = 3.35 min): m/z calcd. for $C_{26}H_{28}N_4O_5$ $[M+H]^+$: 477.2, found 477.2 $[M+H]^+$.

HRMS (ESI) $[M+H]^+$ calcd. for $C_{26}H_{28}N_4O_5$ 477.2132, found 476.2025.

^{1}H NMR (400 MHz, $CDCl_3$) δ 7.53 (d, *J* = 16.1 Hz, 1H), 7.46 (dd, *J* = 8.9, 4.0 Hz, 3H), 7.06 (d, *J* = 16.1 Hz, 1H), 6.88 (d, *J* = 8.7 Hz, 2H), 6.63 (dd, *J* = 9.1, 2.6 Hz, 1H), 6.52 (d, *J* = 2.6 Hz, 1H), 5.54 (s, 2H), 4.06 (q, *J* = 7.0 Hz, 2H), 3.94 (s, 2H), 3.42 (q, *J* = 7.1 Hz, 4H), 1.42 (t, *J* = 7.0 Hz, 3H), 1.22 (t, *J* = 7.1 Hz, 6H).

^{13}C NMR (101 MHz, $CDCl_3$) δ 168.2, 161.1, 159.3, 154.9, 150.3, 140.0, 135.6, 130.3, 128.3, 125.8, 119.6, 118.1, 114.8, 109.4, 108.3, 97.7, 63.6, 59.9, 50.5, 44.9, 15.0, 12.7.

Synthesis of cPlk1i-COOc

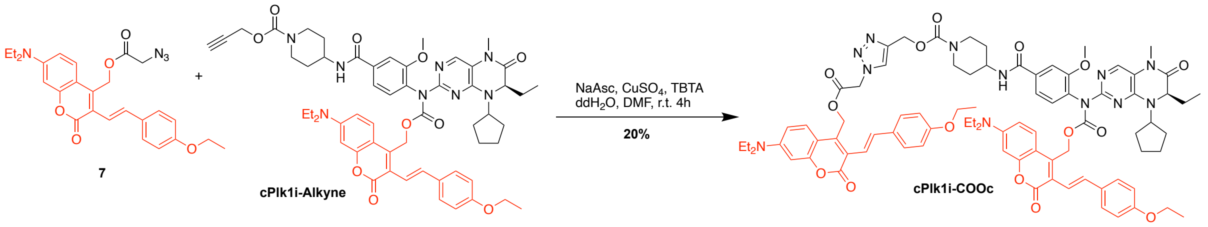

To a solution of sodium ascorbate (20 µL, 500 mM in H_2O, 10 µmol, 1.3 eq.) and $CuSO_4$ (10 µL, 400 mM in H_2O, 4 µmol, 0.5 eq.), TBTA (1 mg 5.3 eq.) was added in a microcentrifuge tube and diluted with 22 µL of water and 100 µL DMF. Then, **cPlk1i-Alkyne** (50 µL, 150 mM in DMF, 7.5 µmol, 1 eq.) was added followed by **7** (50 µL, 150 mM in DMF, 7.5 µmol, 1 eq.). The mixture was shaken at room temperature for 3h in the dark. The crude was diluted with DMF and purified by reverse phase HPLC (50% ACN and 50% H_2O to 100% ACN, without TFA, 16 min) C8 Column. **cPlk1i-COOc** was obtained after lyophilization as a yellow powder (2.3 mg, 1.5 µmol, 20% yield).

LCMS (ESI$^+$, B5, R_t = 3.22 min): m/z calcd. for $C_{82}H_{92}N_{12}O_{15}$ $[M+H]^+$: 1485.7, found 1485.5 $[M+H]^+$.

HRMS (ESI) $[M+H]^+$ calcd. for $C_{82}H_{92}N_{12}O_{15}$ 1485.6884, found 1485.6785.

^{1}H NMR (500 MHz, $CDCl_3$) δ 7.74 (d, *J* = 1.9 Hz, 2H), 7.69 (d, *J* = 9.2 Hz, 1H), 7.45 (d, *J* = 16.1 Hz, 1H), 7.41 (d, *J* = 9.1 Hz, 1H), 7.37 – 7.31 (m, 2H), 7.29 (dd, *J* = 5.5, 1.1 Hz, 2H), 7.22 – 7.15 (m, 4H), 6.94 (d, *J* = 16.2 Hz, 1H), 6.89 (d, *J* = 16.0 Hz, 1H), 6.84 (d, *J* = 8.8 Hz, 2H), 6.77 (d, *J* = 8.8 Hz, 2H), 6.63 (dd, *J* = 9.1, 2.6 Hz, 1H), 6.56 (dd, *J* = 9.2, 2.6 Hz, 1H), 6.49 (d, *J* = 2.6 Hz, 1H), 6.43 (d, *J* = 2.6 Hz, 1H), 6.09 (d, *J* = 7.9 Hz, 1H), 5.57 (s, 1H), 5.47 (s, 2H), 5.30 – 5.11 (m, 4H), 4.13 (dd, *J* = 7.1, 3.5 Hz, 1H), 4.01 (dq, *J* = 18.0, 7.0 Hz, 6H), 3.56 (s, 3H), 3.41 (dq, *J* = 18.0, 7.1 Hz, 8H), 3.26 (s, 3H), 2.85 (t, *J* = 12.7 Hz, 2H), 1.83 (ddd, *J* = 14.2,

7.5, 3.5 Hz, 2H), 1.62 – 1.50 (m, 11H), 1.40 (dt, J = 11.0, 7.1 Hz, 6H), 1.21 (dt, J = 15.6, 7.1 Hz, 13H), 0.91 – 0.79 (m, 3H), 0.75 (t, J = 7.5 Hz, 3H).
^{13}C NMR (126 MHz, $CDCl_3$) δ 166.3, 165.9, 164.2, 161.4, 161.3, 159.4, 159.0, 155.4, 155.1, 154.9, 154.8, 154.5, 153.6, 151.6, 150.4, 150.2, 144.4, 141.7, 139.8, 137.3, 135.6, 135.3, 134.3, 132.7, 130.5, 130.2, 130.1, 129.9, 129.0, 128.3, 128.2, 126.9, 125.7, 125.6, 119.8, 119.0, 118.9, 118.8, 118.7, 117.9, 114.8, 114.6, 110.9, 109.5, 109.4, 108.7, 108.2, 97.7, 97.3, 77.4, 77.2, 76.9, 63.6, 63.6, 62.5, 61.0, 60.5, 59.9, 58.4, 55.7, 50.9, 47.2, 45.0, 44.9, 43.1, 31.8, 30.5, 29.9, 29.7, 28.5, 28.4, 28.2, 27.2, 23.4, 23.2, 15.0, 15.0, 12.7, 12.7, 8.9.

Stability in medium

To assess the stability in medium using LC-MS the compounds were diluted into Fluorobrite + 10% FCS and 10% DMSO at a concentration of 50 μM. The solutions were incubated at 37°C, 5% CO_2 for the indicated time points. An aliquot was taken to measure the LC-MS spectrum. The area under the curve of the absorption spectrum at 260 nm was calculated for the peak corresponding to the caged inhibitor. The area at each time point was normalized to the area at time point 0 hours.

Localization in lysosomes and endosomes

To assess whether the inhibitors accumulate in lysosomes or endosomes HeLa K cells were incubated with the inhibitors and LysoTracker Red (2000x, Invitrogen) for 30 minutes at 37°C. Images were acquired using Nikon Eclipse Ti2-E inverted microscope (Nikon), equipped with Kinetix sCMOS camera (Photometrics), Spectrax Chroma light engine for fluorescence illumination (Lumencor), and an incubation chamber with 37°C, 5% CO2 and controlled humidity (OkoLab). Two-dimensional images were acquired using NIS Elements (Nikon) and 60x Plan Apochromat Lambda objective (NA 1.4, Nikon) with an excitation of 488 nm (for coumarin excitation) and 555 nm (for LysoTracker excitation). The image was focused on a plane with vesicles stained by LysoTracker Red. Analysis was carried out in Fiji (ImageJ). To quantify colocalization a line profile was measured through individual vesicles and the raw intensity along the line profile was plotted for both the green (**cPlk1i** or **cPlk1i-COOc**) channel and the red (LysoTracker Red) channel.

Toxicity assay

To assess the toxicity of the caged and the activated compounds HeLa K cells were seeded in μ-plate 96-well plates (as discussed in main methods). The next day cells were incubated for with the inhibitors at the indicated concentrations. Activation of the inhibitors was performed under the microscope (see Uncaging under the microscope – 2D cell culture). Cells were incubated at 37°C, 5% CO_2 for the indicated times. At the end point cells were treated with Hoechst 33342 (1:2000, Molecular Probes, 10 mg/mL) and propidium iodide (PI, 1:2000, Bio-Techne, Catalog# 5135) for 30 mintues. Images were acquired with IXM confocal automatic microscope (Molecular Device) with 20x water immersion objective (0.95 NA). 6 z-stacks spanning 12 μm. Maximum intensity projection was used for automatic analysis. Image analysis was performed with MetaXpress Custom Module editor software. Briefly, cell and

nuclei masks were created using Hoechst to generate the master object (cell). Dead cells were identified as PI positive. The average number of cells and average number of dead cells was counted for each field of view. The percentage of live cell was calculated. Statistical analysis was performed with GraphPad Prism.

Immunofluorescence and imaging of α-tubulin, γ-tubulin, Plk1 and p676-BubR1

After treatment and activation in 6-well plates cells were incubated for 4-6 hours before fixation. Cells were washed with PBS and fixed with ice cold methanol or fixation medium (20 mM PIPES (pH = 6.8), 10 mM EGTA, 1 mM $MgCl_2$, 0.2% Triton X-100, 4% formaldehyde) for 10 minutes at -20°C or room temperature. After fixation the sample was washed twice with PBS and blocked with PBS 3% BSA. Cells were incubated with anti-α-tubulin (1:1000, DM1A, Sigma), anti-γ-tubulin (1:2000, Wilhelm et al. 2019)[4], anti-ACA human antibody (1:1000, 15-235, ANAWA) and anti-p676-BubR1 rabbit antibody (1:1000, Elowe et al., 2007)[5] for 1 hour or with anti-Plk1 mouse antibody (1:250, ab17057, abcam) over night at 4°C. The sample was washed three times with PBS, 0.01% TritonX-100 before incubation with cross-absorbed secondary anti-mouse or anti-rabbit and anti-human antibodies (1:1000, ThermoFisher Scientific) and DAPI (1:1000) in PBS 3% BSA for 1 hour. Samples were washed three times with PBS and mounted with mowiol. Immunofluorescence images of cells were acquired on an Olympus DeltaVision wide-field microscope (GE Healthcare) equipped with a DAPI/FITC/TRITC/Cy5 filter set (Chroma Technology Corp.) and a Coolsnap HQ2 CCD camera (Roper Scientific) running Softworx 6.5.2 (GE Healthcare). The kinetochores were imaged using a 60x 1.4 NA objective with 0.2 µm Z-stacks spanning 12 µm. 3D images were deconvolved using Softworx 6.5.2 (GE Healthcare) in conservative mode. Maximum intensity projection was used for analysis. Positive p676-BubR1 cells were identified by having a signal at the kinetochore with the brightness set to 4000. Mitotic spindle phenotypes were scored based on the α-tubulin staining. γ-tubulin intensity was quantified with a 6x6 pixel ROI at the spindle poles and normalized to the intensity in the cytoplasm. Plk1 intensity was quantified with a 6x6 pixel ROI at 5 different kinetochores per cell and normalized to the fluorescence in the cytoplasm. Statistical analysis was performed with GraphPad Prism.

Supplementary References

1. Chen, L. *et al.* BRD4 Structure–Activity Relationships of Dual PLK1 Kinase/BRD4 Bromodomain Inhibitor BI-2536. *ACS Medicinal Chemistry Letters* **6**, 764–769 (2015).
2. Lin, Q. *et al.* Coumarin Photocaging Groups Modified with an Electron-Rich Styryl Moiety at the 3-Position: Long-Wavelength Excitation, Rapid Photolysis, and Photobleaching. *Angewandte Chemie International Edition* **57**, 3722–3726 (2018).

3. O'brien, A. G., Lévesque, F. & Seeberger, P. H. Continuous flow thermolysis of azidoacrylates for the synthesis of heterocycles and pharmaceutical intermediates. *Chemical Communications* **47**, 2688–2690 (2011).
4. Wilhelm, T. *et al.* Mild replication stress causes chromosome mis-segregation via premature centriole disengagement. *Nat Commun* **10**, 3585 (2019).
5. Elowe, S., Hümmer, S., Uldschmid, A., Li, X. & Nigg, E. A. Tension-sensitive Plk1 phosphorylation on BubR1 regulates the stability of kinetochore microtubule interactions. *Genes & development* **21**, 2205–2219 (2007).
