# Supplementary material for: Spatio-temporal control of mitosis using light via a Plk1 inhibitor caged for activity and cellular permeability
Source: Nat Commun. 2025 Feb 19;16:1599. doi: 10.1038/s41467-025-56746-5 (PMC11840123; doi:10.1038/s41467-025-56746-5)
Supplement: Supplementary file 2 — Description of Additional Supplementary Files [file 41467_2025_56746_MOESM2_ESM.pdf]

Description of Additional Supplementary Files

File Name: Supplementary Movie 1

Description: Spheroids of stably expressing H2BmCherry (white) cells treated with DMSO. Time points indicated in hh:min. The top left quadrant is exposed to light. Images were taken every 15 minutes for 12 hours.

File Name: Supplementary Movie 2 and 3

Description: Spheroids of stably expressing H2B-mCherry (white) cells treated with cPlk1iCOOc (5 μM). Time points indicated in hh:min. The top left quadrant is exposed to light. Images were taken every 15 minutes for 12 hours.
