# Supplementary material for: Spatio-temporal control of mitosis using light via a Plk1 inhibitor caged for activity and cellular permeability
Source: Nat Commun. 2025 Feb 19;16:1599. doi: 10.1038/s41467-025-56746-5 (PMC11840123; doi:10.1038/s41467-025-56746-5)
Supplement: Supplementary file 6 — Reporting Summary [file 41467_2025_56746_MOESM6_ESM.pdf]

nature portfolio

Corresponding author(s): Nicolas Winssinger, Monica Gotta

Last updated by author(s): Jan 8, 2025

Reporting Summary

Nature Portfolio wishes to improve the reproducibility of the work that we publish. This form provides structure for consistency and transparency in reporting. For further information on Nature Portfolio policies, see our Editorial Policies and the Editorial Policy Checklist.

Statistics

For all statistical analyses, confirm that the following items are present in the figure legend, table legend, main text, or Methods section.

n/a	Confirmed	
☐	☒	The exact sample size (*n*) for each experimental group/condition, given as a discrete number and unit of measurement
☐	☒	A statement on whether measurements were taken from distinct samples or whether the same sample was measured repeatedly
☐	☒	The statistical test(s) used AND whether they are one- or two-sided<br>*Only common tests should be described solely by name; describe more complex techniques in the Methods section.*
☒	☐	A description of all covariates tested
☐	☒	A description of any assumptions or corrections, such as tests of normality and adjustment for multiple comparisons
☐	☒	A full description of the statistical parameters including central tendency (e.g. means) or other basic estimates (e.g. regression coefficient) AND variation (e.g. standard deviation) or associated estimates of uncertainty (e.g. confidence intervals)
☐	☒	For null hypothesis testing, the test statistic (e.g. *F*, *t*, *r*) with confidence intervals, effect sizes, degrees of freedom and *P* value noted<br>*Give P values as exact values whenever suitable.*
☒	☐	For Bayesian analysis, information on the choice of priors and Markov chain Monte Carlo settings
☒	☐	For hierarchical and complex designs, identification of the appropriate level for tests and full reporting of outcomes
☒	☐	Estimates of effect sizes (e.g. Cohen's *d*, Pearson's *r*), indicating how they were calculated

Our web collection on statistics for biologists contains articles on many of the points above.

Software and code

Policy information about availability of computer code

Data collection	All instruments were controlled with commercial software provided by the manufacturer. 1H and 13C NMR spectra were recorded on Bruker Avance 3 HD NMR spectrometer 400 MHz and Bruker 500 UltraShield NMR spectrometer 500 MHz. LC-MS spectra were recorded using a DIONEX Ultimate 3000 UHPLC coupled with a Thermo LCQ Fleet Mass Spectrometer System (electrospray ionization (ESI)). Absorption spectra were measured using a Jasco v-650 spectrophotometer. Fluorescence emission was measured using Molecular Devices Spectra Max M5 spectrometer or a TECAN SPARK multimode microplate reader. Uptake of the inhibitor was measured with an A1r microscope (Nikon) equipped with 405 nm 100 mW laser, 488 nm 50 mW laser, excitation filter 472/30 nm and emission filter 520/35 nm and a 640 nm 40 mW laser, excitation filter 628/40 nm and emission filter 692/40 nm and a DU4 detector, using a Plan Apo VC 60x (NA 1.4) objective. For uncaging and imaging the IXM confocal automatic microscope (Molecular Device) with 10 x air objective and 20x water immersion objective (0.95 NA) was used. Live-cell imaging was performed on a temperature- and CO2-controlled Zeiss LSM 980 scanning confocal microscope equipped with a Multialkali-PTM detector, and a Plan-ApoChromat 10x/0.45 NA air objective. Lysosomes were acquired using Nikon Eclipse Ti2-E inverted microscope (Nikon), equipped with Kinetix sCMOS camera (Photometrics), Spectrax Chroma light engine for fluorescence illumination (Lumencor), and an incubation chamber with 37°C, 5% CO2 and controlled humidity (OkoLab). Two-dimensional images were acquired using NIS Elements (Nikon) and 60x Plan Apochromat Lambda objective (NA 1.4, Nikon). Immunofluorescence images were acquired on an Olympus DeltaVision wide-field microscope (GE Healthcare) equipped with a DAPI/FITC/TRITC/Cy5 filter set (Chroma Technology Corp.) and a Coolsnap HQ2 CCD camera (Roper Scientific) running Softworx 6.5.2 (GE Healthcare). The kinetochores were imaged using a 60x 1.4 NA objective. Images of the lysosomes were acquired using Nikon Eclipse Ti2-E inverted microscope (Nikon), equipped with Kinetix sCMOS camera (Photometrics), Spectrax Chroma light engine for fluorescence illumination (Lumencor), and an incubation chamber with 37°C, 5% CO2 and controlled humidity (OkoLab). Two-dimensional images were acquired using NIS Elements (Nikon) and 60x Plan Apochromat Lambda objective (NA 1.4, Nikon) with an excitation of 488 nm (for coumarin excitation) and 555 nm (for LysoTracker excitation).
Data analysis	For the NMR acquisition the software used is Bruker IconNMR 5.0.10.Build19, or TopSpin 3.6.2 and the spectra is analyzed with MestReNova v

12.0.1-20560. Microscopy images were processed and analyzed in Fiji (ImageJ 2.14.0) or MetaExpress (6.5.3). Statistical analysis was performed with GraphPad Prism 10 or RStudio (4.4.1). Figures were assembled in Adobe Illustrator 2023. Chemical structures were drawn in ChemDraw 22.0.0 (PerkinElmer). The co-crystal structure (PBD 2RKU) was made in PyMOL with the data from Kothe et al. 2007.

For manuscripts utilizing custom algorithms or software that are central to the research but not yet described in published literature, software must be made available to editors and reviewers. We strongly encourage code deposition in a community repository (e.g. GitHub). See the Nature Portfolio guidelines for submitting code & software for further information.

Data

Policy information about availability of data

All manuscripts must include a data availability statement. This statement should provide the following information, where applicable:

- Accession codes, unique identifiers, or web links for publicly available datasets
- A description of any restrictions on data availability
- For clinical datasets or third party data, please ensure that the statement adheres to our policy

The authors declare that all data supporting the findings of this study are available in the article, its supplementary files, as well as in the source data file. All raw data related to main and supplementary figures are deposited in the Yareta repository (10.26037/yareta:os6ctilkh5d4hgqrpbdu4lk45u).

Research involving human participants, their data, or biological material

Policy information about studies with human participants or human data. See also policy information about sex, gender (identity/presentation), and sexual orientation and race, ethnicity and racism.

Reporting on sex and gender	N/A
Reporting on race, ethnicity, or other socially relevant groupings	N/A
Population characteristics	N/A
Recruitment	N/A
Ethics oversight	N/A

Note that full information on the approval of the study protocol must also be provided in the manuscript.

Field-specific reporting

Please select the one below that is the best fit for your research. If you are not sure, read the appropriate sections before making your selection.

☒ Life sciences ☐ Behavioural & social sciences ☐ Ecological, evolutionary & environmental sciences

For a reference copy of the document with all sections, see nature.com/documents/nr-reporting-summary-flat.pdf

Life sciences study design

All studies must disclose on these points even when the disclosure is negative.

Sample size	Sample sizes were chosen according to commonly used and accepted standards in the field.
Data exclusions	Spheroids that moved or turned during the acquisition were excluded from the analysis. Images and movies out of focus were excluded from the analysis.
Replication	Reproducibility of experiments was confirmed. Key findings were replicated across independent experiments.
Randomization	Randomization was not suitable for this study as the experimentalist had to prepare the samples and treatments.
Blinding	Blinding was not suitable to this study as the biological samples all have distinct treatments and the experimentalists had to prepare the samples and treatments. However, all data analysis was streamlined and automated whenever possible such that no bias is invoked.

Reporting for specific materials, systems and methods

We require information from authors about some types of materials, experimental systems and methods used in many studies. Here, indicate whether each material, system or method listed is relevant to your study. If you are not sure if a list item applies to your research, read the appropriate section before selecting a response.

Materials & experimental systems

n/a	Involved in the study
[ ]	[x] Antibodies
[ ]	[x] Eukaryotic cell lines
[x]	[ ] Palaeontology and archaeology
[x]	[ ] Animals and other organisms
[x]	[ ] Clinical data
[x]	[ ] Dual use research of concern
[x]	[ ] Plants

Methods

n/a	Involved in the study
[x]	[ ] ChIP-seq
[ ]	[x] Flow cytometry
[x]	[ ] MRI-based neuroimaging

Antibodies

Antibodies used	anti-α-tubulin primary antibody (1:1000, DM1A, Sigma), anti-γ-tubulin primary antibody (1:2000, Wilhelm et al. 2019), anti-BubR1 mouse primary antibody (1:20, ABCD antibodies, AW952); anti-mouse AlexaFluor 647 (1:200, Jackson Immunoresearch) secondary antibody; anti-ACA human antibody (1:1000, 15-235, ANAWA); anti-p676-BubR1 rabbit antibody (1:1000, Elowe et al., 2007); anti-Plk1 mouse antibody (1:250, ab17057, abcam); cross-absorbed secondary anti-mouse or anti-rabbit and anti-human antibodies (1:1000, ThermoFisher Scientific).
Validation	anti-α-tubulin primary antibody (1:1000, DM1A, Sigma), anti-γ-tubulin primary antibody (1:2000, Wilhelm et al. 2019), anti-BubR1 mouse primary antibody (1:20, ABCD antibodies, AW952); DeLuca, eLife, 2021 (PMID: 34970967); anti-ACA human antibody (1:1000, 15-235, ANAWA); anti-p676-BubR1 rabbit antibody (1:1000, Elowe et al., 2007); anti-Plk1 mouse antibody (1:250, ab17057, abcam).

Eukaryotic cell lines

Policy information about cell lines and Sex and Gender in Research

Cell line source(s)	HeLa K cells (atcc, CCL-2), HeLa H2B-mCherry/MTS-GFP cells (Kind gift of Izabela Sumara)
Authentication	Cell lines were not authenticated.
Mycoplasma contamination	Cells were not specifically tested for mycoplasma.
Commonly misidentified lines (See ICLAC register)	The cell lines used were not listed in the ICLAC register.

Plants

Seed stocks	N/A
Novel plant genotypes	N/A
Authentication	N/A

Flow Cytometry

Plots

Confirm that:

- [x] The axis labels state the marker and fluorochrome used (e.g. CD4-FITC).
- [x] The axis scales are clearly visible. Include numbers along axes only for bottom left plot of group (a 'group' is an analysis of identical markers).
- [x] All plots are contour plots with outliers or pseudocolor plots.
- [x] A numerical value for number of cells or percentage (with statistics) is provided.

Methodology

Sample preparation	see Materials and Methods "Cell cycle analysis by flow cytometry"

Instrument	Accuri c6 flow cytometer (BD Bioscience)
Software	BD Accuri C6 software, FlowJoTM software
Cell population abundance	20000 cells were analyzed per sample
Gating strategy	See Supplementary Fig. 5.

☒ Tick this box to confirm that a figure exemplifying the gating strategy is provided in the Supplementary Information.
